# Biomechanical Integrity Score of the Female Pelvic Floor for Stress Urinary Incontinence

**DOI:** 10.1007/s00192-024-05797-1

**Published:** 2024-05-13

**Authors:** Peter Takacs, Dávid Rátonyi, Erzsébet Koroknai, Heather van Raalte, Vincent Lucente, Vladimir Egorov, Zoard Tibor Krasznai, Bence Kozma

**Affiliations:** 1 Department of Obstetrics and Gynecology, Division of Female Pelvic Medicine and Reconstructive Surgery, Eastern Virginia Medical School, Norfolk, VA USA; 2 Faculty of Medicine, Department of Obstetrics and Gynecology, University of Debrecen, Nagyerdei Krt 98, 4032 Debrecen, Hungary; 3 The Institute for Female Pelvic Medicine & Reconstructive Surgery, Allentown, PA USA; 4 Princeton Urogynecology, Princeton, NJ USA; 5 Advanced Tactile Imaging, Ewing, NJ USA

**Keywords:** Stress urinary incontinence, Tissue elasticity, Pelvic support, Pelvic muscle strength, Muscle mobility, Biomechanical Integrity score

## Abstract

**Introduction and hypothesis:**

This study is aimed at developing and validating a new integral parameter, the Biomechanical Integrity score (BI-score) of the female pelvic floor for stress urinary incontinence conditions.

**Methods:**

A total of 130 subjects were included in the observational cohort study; 70 subjects had normal pelvic floor conditions, and 60 subjects had stress urinary incontinence (SUI). A Vaginal Tactile Imager (VTI) was used to acquire and automatically calculate 52 biomechanical parameters for eight VTI test procedures (probe insertion, elevation, rotation, Valsalva maneuver, voluntary muscle contractions in two planes, relaxation, and reflex contraction). Statistical methods were applied (*t* test, correlation) to identify the VTI parameters sensitive to the pelvic SUI conditions.

**Results:**

Twenty-seven parameters were identified as statistically sensitive to SUI development. They were subdivided into five groups to characterize tissue elasticity (group 1), pelvic support (group 2), pelvic muscle contraction (group 3), involuntary muscle relaxation (group 4), and pelvic muscle mobility (group 5). Every parameter was transformed to its standard deviation units using the dataset for normal pelvic conditions, similar to the T-score for bone density. Linear combinations with specified weights led to the composition of five component parameters for groups 1–5 and to the BI-score in standard deviation units. The *p* value for the BI-score has *p* = 4.0 × 10^–28^ for SUI versus normal conditions.

**Conclusions:**

Quantitative transformations of the pelvic tissues, support structures, and functions under diseased conditions may be studied with the SUI BI-score in future research and clinical applications.

## Introduction

Urinary incontinence (UI) is a prevalent condition, affecting more than 1 in 3 women at some point in their lives [1]. More than 20 million women in the USA are affected by UI, and, based on current demographic trends, this number is expected to increase by more than 50% in the coming decades [2]. UI leads to physical, emotional, and social distress, significantly limiting one's lifestyle and ability to engage in work-related activities [3]. The three main types of urinary incontinence are stress, urge, and mixed. Stress urinary incontinence (SUI) occurs when unintended urine leakage appears during coughing, sneezing, or physical exertion [4]. SUI is the most common type overall, but mixed urinary incontinence is the most common type among older women [5]. The prevalence of SUI (defined as any symptoms in the previous year) in adult women is about 46% [6]. Even though UI is widespread, it often goes unnoticed and unreported, with fewer than 40% of affected women seeking medical attention for this issue [7]. It is, therefore, important for health care providers to accurately screen for and diagnose SUI. A thorough medical history is essential for correct diagnosis and assessment. However, it is often difficult to obtain accurate data as patients may be reluctant to talk about their urinary symptoms or be unable to give accurate information about the exact course of the disease, although this information is crucial as it can determine the treatment choice. For this reason, the various questionnaires are helpful in accurately assessing symptoms and disease severity. The Urinary Distress Inventory 6 and Incontinence Impact Questionnaire 7 are most commonly used to assess the symptoms of SUI, whereas the Medical, Epidemiologic, and Social Aspects of Aging (MESA) and the Patient Global Impression of Severity tools are used to assess the severity of the disease [8, 9]. However, further tests may be needed if the answers to the questionnaire are inconclusive. In addition to urinalysis and pelvic examination, urodynamic testing is the cornerstone of diagnosis. In many cases, urodynamic testing is mildly uncomfortable for the patient, but it is an expensive and highly specialized test unsuitable for screening. A common feature of these studies is that they provide little information about etiological factors. The pathology of SUI is complex and influenced by multiple factors. There is substantial evidence suggesting the involvement of bladder neck and urethral incompetence [10, 11]. Additionally, there are indications of compromised urethral support and levator ani muscle function. Therefore, there is a need for standardized measurement approaches to generate more robust and conclusive evidence in this regard [12].

The Vaginal Tactile Imager (VTI) was developed to provide a biomechanical mapping of the pelvic floor with a vaginal probe [13]. A set of new clinical markers/parameters has been proposed for the biomechanical characterization of pelvic floor conditions [14]. This set included 52 parameters automatically calculated as a result of completing eight examination procedures (tests). The Biomechanical Integrity score (BI-score) for pelvic organ prolapse (POP) was proposed [15]. In order to make biomechanical mapping in urogynecology more accessible and valuable, further work is required on developing a shorter list of easily understandable and practical biomechanical parameters for SUI characterization.

This article is aimed at reporting the development and validation of a new integral parameter, the BI-score, for characterizing the female pelvic floor under SUI.

## Materials and Methods

### Definitions

Tactile imaging is a medical imaging modality that translates the sense of touch into a digital image [13]. The tactile image is a function of P(x,y,z), where P is the pressure on the soft-tissue surface under applied deformation, and x, y, and z are the coordinates where P was measured. The tactile image is a pressure map on which the direction of tissue deformation must be specified.

Functional tactile imaging translates muscle activity into the dynamic pressure pattern P(x,y,t) for an area of interest, where t is time and x and y are coordinates where the pressure P was measured. It may include a muscle voluntary contraction, an involuntary reflex contraction, involuntary relaxation, and specific maneuvers.

$$Biomechanical\;mapping\:=\:tactile\;imaging\:+\:functional\;tactile\;imaging$$

A tactile imaging probe has a pressure sensor array mounted on its face that acts in similar manner to human fingers during a clinical examination, deforming the soft tissue and detecting the resulting changes in the pressure pattern on the surface. The sensor head is moved against or over the surface of the tissue to be studied, and the pressure response is measured at multiple locations along the tissue. The results are used to generate images that show pressure distribution over the area of the tissue under study. The tactile image P(x,y,z) reveals tissue or organ anatomy and elasticity distribution [16, 17].

### Vaginal Tactile Imager

**Fig. 1:**
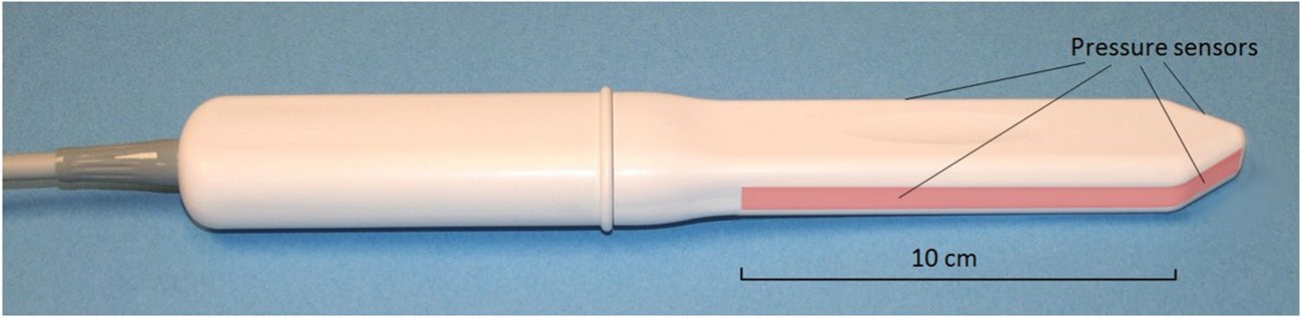
Vaginal probe. Pressure sensors are aligned on the outer surfaces of the probe (highlighted in the image), with permission from Advanced Tactile Imaging, Inc

The VTI, model 2S, was used for biomechanical mapping of the pelvic floor. As shown in Fig. 1, the VTI probe is equipped with 96 pressure (tactile) sensors spaced consecutively on both sides of the probe, an orientation sensor, and temperature controllers to provide the probe temperature close to a human body before the examination. During the clinical procedure, the probe is used to acquire pressure responses from two opposite vaginal walls (anterior–posterior and left–right) along the vagina. The VTI data are sampled from the probe sensors and presented on the VTI display in real time. The resulting pressure maps (tactile images) of the vagina integrate all the acquired pressure and positioning data for each pressure-sensing element during vaginal wall deformation and pelvic muscle contraction. Lubricating gel is used for patient comfort. It also provides reproducible boundary/contact conditions with deformed tissues.

The VTI examination procedure consists of eight tests. The spatial gradients ∂P(x, y)/∂y (changes of pressure along the tissue deformation per 1 mm) for the anterior and posterior compartments are calculated within the acquired tactile images in tests 1 and 2; the y-coordinate is directed orthogonally from the vaginal channel coming through the anterior–posterior compartments, and the x-coordinate is located on the vaginal channel.

The VTI probe is calibrated with reference pressure sensors (Honeywell Inc., Charlotte, NC, USA) immediately before every subject examination. The VTI absolute measurement accuracy is as follows: ± 0.2 kPa within the 10 kPa range, ± 0.5 kPa at 25 kPa, and ± 1.0 kPa at 60 kPa. The VTI relative pressure measurement accuracy ranges from ± 0.05 kPa to ± 0.1 kPa. The intra- and inter-observer reproducibility of vaginal tactile imaging was reported earlier [18]. Intra-observer intraclass correlation coefficients (ICCs) were found in the range from 0.80 (test 8: cough) to 0.92 (test 3: rotation) with an average value of 0.87. Inter-observer ICCs ranged from 0.73 (test 2 elevation pressure and test 8 cough) to 0.92 (test 3 rotation), with an average value of 0.82. Intra-observer limits of agreement ranged from ± 11.3% (test 1) to ± 19.0%% (test 8) with an average value of ± 15.1%. Inter-observer limits of agreement ranged from ± 12.0% (test 5 voluntary contraction) to ± 26.7% (test 2 elevation) with an average value of ± 18.4%. These numbers lead to the projection of reproductivity for the BI-score and its components in the range from ± 0.1 to ± 0.2 standard deviation. Improved inter-observer reproducibility is possible through additional operator training and consistency in the VTI examination technique. The VTI pressure measurement resolution is 10 Pa. The VTI absolute measurement accuracy for probe orientation is ± 0.5 °C and ± 0.1 °C for measuring the temperature inside the probe on the surface of the pressure sensors. The tactile images and muscle contraction patterns are visualized with a resolution of 1.0 mm [13].

### Biomechanical Parameters

The complete list of 52 VTI biomechanical parameters, their interpretation, and anatomical assignments of the targeting/contributing pelvic structures into the specified parameters are presented in a previous publication [14].

The parameters listed in Table 1 have different units (see the fourth column in Table 1). The next step was to bring all the selected parameters to uniform units to allow their arithmetic combination. Among various possible options, the preference for the units of standard deviation was provided (see the explanation pertaining to such selection in the Discussion section). All VTI data were transformed according to Eq. 1 below.

**Table 1:** Vaginal Tactile Imager (*VTI*) biomechanical parameters demonstrating sensitivity to stress urinary incontinence (*SUI*) conditions

VTI parameter number	VTI test number	Parameter abbreviation	Units	BI-score component	Parameter weight	Norm mean (*n* = 70)	Norm SD (*n* = 70)	SUI mean (*n* = 60)	SUI, SD (*n* = 60)	100%*(SUI-norm)/norm	Norm vs SUI *p*-value
2	1	Work	mJ	Elasticity	0.143	55.18	22.78	23.39	10.88	−57.6	1.8E-17
3	1	Gmax_a	kPa/mm	Elasticity	0.143	2.30	1.44	0.81	0.55	−64.9	6.8E-12
4	1	Gmax_p	kPa/mm	Elasticity	0.143	1.55	0.82	0.63	0.47	−59.4	3.7E-12
6	1	Pmax_p	kPa	Elasticity	0.143	23.87	11.12	9.96	6.13	−58.3	2.1E-14
7	2	P1max_a	kPa	Support	0.125	35.89	22.62	7.63	5.67	−78.7	2.5E-16
8	2	P2max_a	kPa	Support	0.125	12.01	5.41	4.91	2.94	−59.1	1.8E-15
10	2	P1max_p	kPa	Support	0.125	13.81	6.10	5.23	3.04	−62.2	1.7E-17
11	2	P2max_p	kPa	Support	0.125	11.60	4.36	5.38	2.82	−53.6	1.9E-16
12	2	P3max_p	kPa	Support	0.125	7.33	3.55	4.30	2.55	−41.3	1.9E-07
13	2	G1max_a	kPa/mm	Support	0.125	1.38	1.35	0.54	0.70	−61.1	2.5E-05
14	2	G2max_a	kPa/mm	Support	0.125	0.39	0.39	0.23	0.26	−41.8	6.2E-03
17	2	G2max_p	kPa/mm	Support	0.125	0.38	0.20	0.21	0.27	−43.7	8.9E-05
21	3	Fs	*N*	Elasticity	0.143	2.34	0.93	0.95	0.51	−59.4	2.1E-18
22	3	P1_l	kPa	Elasticity	0.143	8.96	3.26	4.41	2.47	−50.7	8.3E-15
24	3	P3_r	kPa	Elasticity	0.143	9.88	4.59	4.84	2.21	−51.1	2.4E-12
27	4	dL_a	mm	Mobility	0.500	4.88	5.52	1.95	7.09	−60.1	9.1E-03
28	4	dF_p	*N*	Contraction	0.125	0.80	0.68	1.37	0.84	71.3	3.9E-05
32	5	dPmax_a	kPa	Contraction	0.125	24.43	15.27	13.97	13.21	−42.8	6.3E-05
33	5	Pmax_a	kPa	Contraction	0.125	49.38	19.62	19.66	14.57	−60.2	6.2E-17
34	5	dF_p	*N*	Contraction	0.125	1.55	0.76	1.19	0.80	−23.0	1.1E-02
36	5	Pmax_p	kPa	Contraction	0.125	22.25	7.41	12.91	6.85	−42.0	1.4E-11
39	6	Pmax_r	kPa	Contraction	0.125	13.96	5.90	8.34	6.01	−40.2	3.7E-07
40	6	dF_l	*N*	Contraction	0.125	0.86	0.47	0.58	0.46	−31.7	1.2E-03
44	7	dpcdt_a	%/s	Relaxation	0.500	−3.63	4.91	−9.17	6.77	152.8	3.7E-07
46	7	dpcdt_p	%/s	Relaxation	0.500	−4.16	3.69	−9.63	6.80	131.5	5.6E-08
47	8	dF_a	*N*	Contraction	0.125	1.52	0.84	1.95	0.91	28.8	5.2E-03
52	8	dL_p	mm	Mobility	0.500	3.63	4.74	7.23	7.27	99.4	9.2E-04

^a^Parameter numbering as in Egorov et al. [15]

1 $${Psd}_{n}^{i}=({Po}_{n}^{i}- {Pa}_{n})/{SD}_{n}$$

**Fig. 2:**
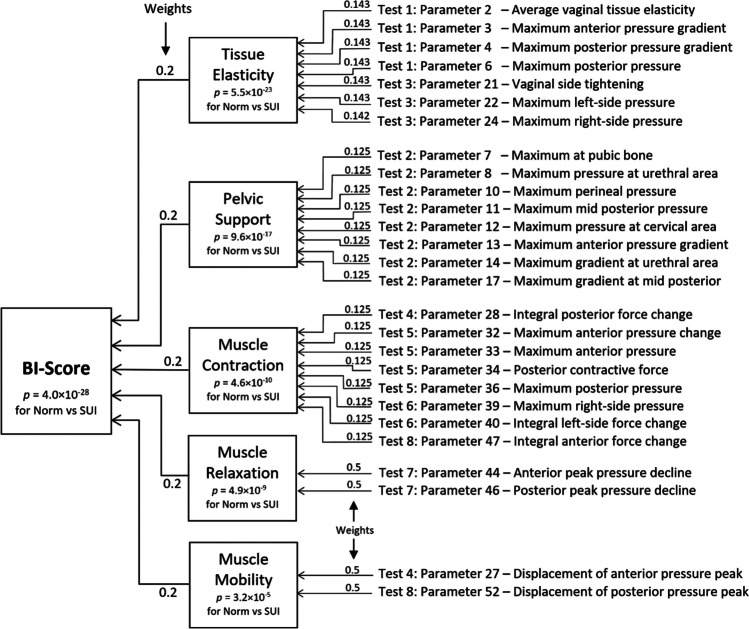
A diagram illustrating the composition of stress urinary incontinence (*SUI*) Biomechanical Integrity (*BI*)-score from five components and Vaginal Tactile Imager parameters contributing to these components with specific weights

Here, ${Po}_{n}^{i}$ is an original value of the *n*-parameter for the *i*-subject; ${Pa}_{n}$ is an arithmetic average of the *n*-parameter for subjects aged 18–39 years in the group with a normal pelvis (70 subjects); ${SD}_{n}$ is a standard deviation for the *n*-parameter for 70 subjects in the group with a normal pelvis, and ${Psd}_{n}^{i}$ is the transformed value of the *n*-parameter for the *i*-subject in units of standard deviation. Now, we can combine the parameters expressed in units of standard deviation using a linear operation of addition. First, the 27 selected parameters were subdivided into five groups. We may call them by the five components to characterize: tissue elasticity (component 1), pelvic support (component 2), pelvic muscle contraction (component 3), muscle relaxation (component 4), and muscle mobility (component 5; Fig. 2). Component 1 comprises seven parameters with weights of 0.143, component 2 comprises eight parameters with weights of 0.125, component 3 consists of eight parameters with weights of 0.125, component 4 comprises two parameters with a weight of 0.5, and component 5 consists of two parameters with a weight of 0.5. It is crucial that VTI parameters 28 and 52 were used with negative signs because they have increased values at SUI versus normal conditions (Table 1). Finally, these five components create the BI-score with equal weights of 0.2, as shown in Fig. 2.

### Study Population

The analyzed dataset in this study includes subjects with normal pelvic floor and SUI from two VTI clinical studies with identical VTI examination procedures. The subjects were examined with the VTI in the scope observational cohort studies completed from May 2022 to June 2023 (study 1) and June 2020 to September 2021 (study 2). Similar clinical protocols were approved by the Institutional Review Board (study 1: Western IRB and local IRB as required; study 2: Scientific and Research Ethics Committee of Hungary: 2876–13/2022/EÜIG), and written informed consent was obtained from all the subjects enrolled in the studies. Table 2 presents the mean and standard deviation for the subject age, parity, weight, and height separately for the normal and POP groups. The VTI examination data for the eight tests were obtained and recorded at the time of the scheduled urogynecology visits [14]. All the analyzed subjects needed not to have had any prior pelvic surgery.

**Table 2:** Demographic data for the studied groups

	Norm mean (*n* = 70)	Norm SD (*n* = 70)	SUI mean (*n* = 60)	SUI SD (*n* = 60)
Patient age, years	27.7	5.5	54.6	12.2
Patient parity	0.5	1.1	1.9	0.7
Patient weight, kg	71.1	22.5	73.8	14.7
Patient height, cm	163	8	163	7

*SD* standard deviation, *SUI* stress urinary incontinence

The total study workflow comprised the following steps:

1. Recruiting women who did not previously have pelvic surgery and had normal pelvic floor conditions (no SUI) or had SUI measured by the Medical, Epidemiologic, and Social Aspects of Aging (MESA) questionnaire [8]
2. Acquiring clinical diagnostic information related to the cases included in the study by standard clinical means
3. Performing a VTI examination in a lithotomy position
4. Analyzing VTI data

Study exclusion criteria were active infection or ulceration within the vagina; presence of a vaginal septum; active cancer of the colon, rectum wall, cervix, vaginal, uterus, or bladder; ongoing radiation therapy for pelvic cancer; impacted stool; significant pre-existing pelvic pain, including levator ani syndrome, severe vaginismus, or vulvodynia; severe hemorrhoids; significant circulatory or cardiac conditions that could cause excessive risk from the examination, as determined by the attending physician; and current pregnancy. In study 2, which targets normal pelvic conditions, one inclusion criterion was modified to women aged 18–39 years, and two exclusion criteria were added as follows: the woman is a regular patient visiting the urogynecology clinic (two or more times during the last year); and cognitive impairment.

### Statistical Methods

A total of 52 biomechanical parameters were calculated automatically by the VTI software version 2023.66.1.0 per each of the 130 analyzed VTI examination data. The two-sample *t* test (*p* < 0.01) was employed to test the null hypothesis that the data in normal and SUI groups have equal means and equal variances. The Bonferroni method was used to adjust for multiple comparisons, changing the commonly accepted statistical *p* value from 0.05 to 0.01. The alternative hypothesis is that the data in these groups come from populations with unequal means. The *p* values for testing the hypothesis were calculated. Pearson's linear correlation coefficients (*r*) were calculated among 52 VTI parameters, each parameter against all other 51 parameters.

To visually evaluate the analyzed data distributions, we used notched boxplots showing a confidence interval for the median value (central vertical line), and 25% and 75% quartiles [19]. The spacing between the different parts of the box helps to compare variance. The boxplot also determines skewness (asymmetry) and outliers (cross). The statistical functions of MATLAB, version R2022b (MathWorks, MA, USA), were used for the data analysis.

## Results

In this study of 130 subjects, 70 subjects had normal pelvic floor conditions, and 60 subjects had SUI according to MESA score. The mean subject age and parity in the normal and SUI groups are significantly different: 27.7 versus 54.6 years old and 0.5 versus 1.9 respectively. The mean subject weight and height are the same in both groups (Table 2). The last column in Table 1 brings *p* values for the two-sample *t* tests (normal versus SUI). The *p* values for the VTI parameters are found in the range of 2.1 × 10 − 18 to 1.1 × 10 − 2, with most *p* values being < 1.0 × 10 − 5. The *p* value for the BI-score has *p* = 4.0 × 10 − 28 for the two analyzed groups.

**Fig. 3:**
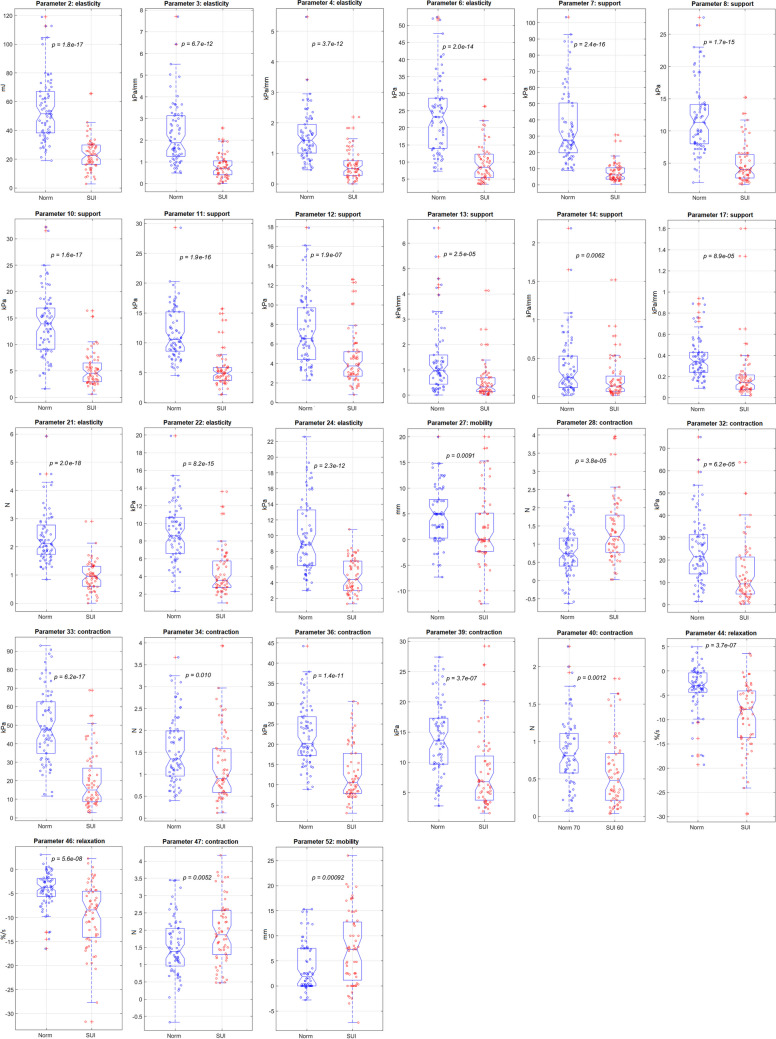
Boxplots for 27 Vaginal Tactile Imager parameters that demonstrate statistically significant sensitivity to stress urinary incontinence (*SUI*) conditions and do not correlate highly with each other

We first aimed to select VTI parameters with significant changes at SUI versus the normal pelvic conditions during the statistical analysis. Two specific quantitative criteria were imposed on such selection: a *t* test *p* < 0.01 for the dataset of 60 SUI cases against the dataset for 70 normal cases; and a correlation coefficient *r* < 0.85 with all other parameters. The first criterion passed 43 parameters; both the first and second 27 parameters. Figure 3 presents the boxplots, and Table 1 shows the numerical data for these selected 27 VTI parameters responsive to SUI and not highly correlated with each other. For consistency, the numbering of the VTI parameters in this article is kept exactly as in earlier publications [14].

**Fig. 4:**
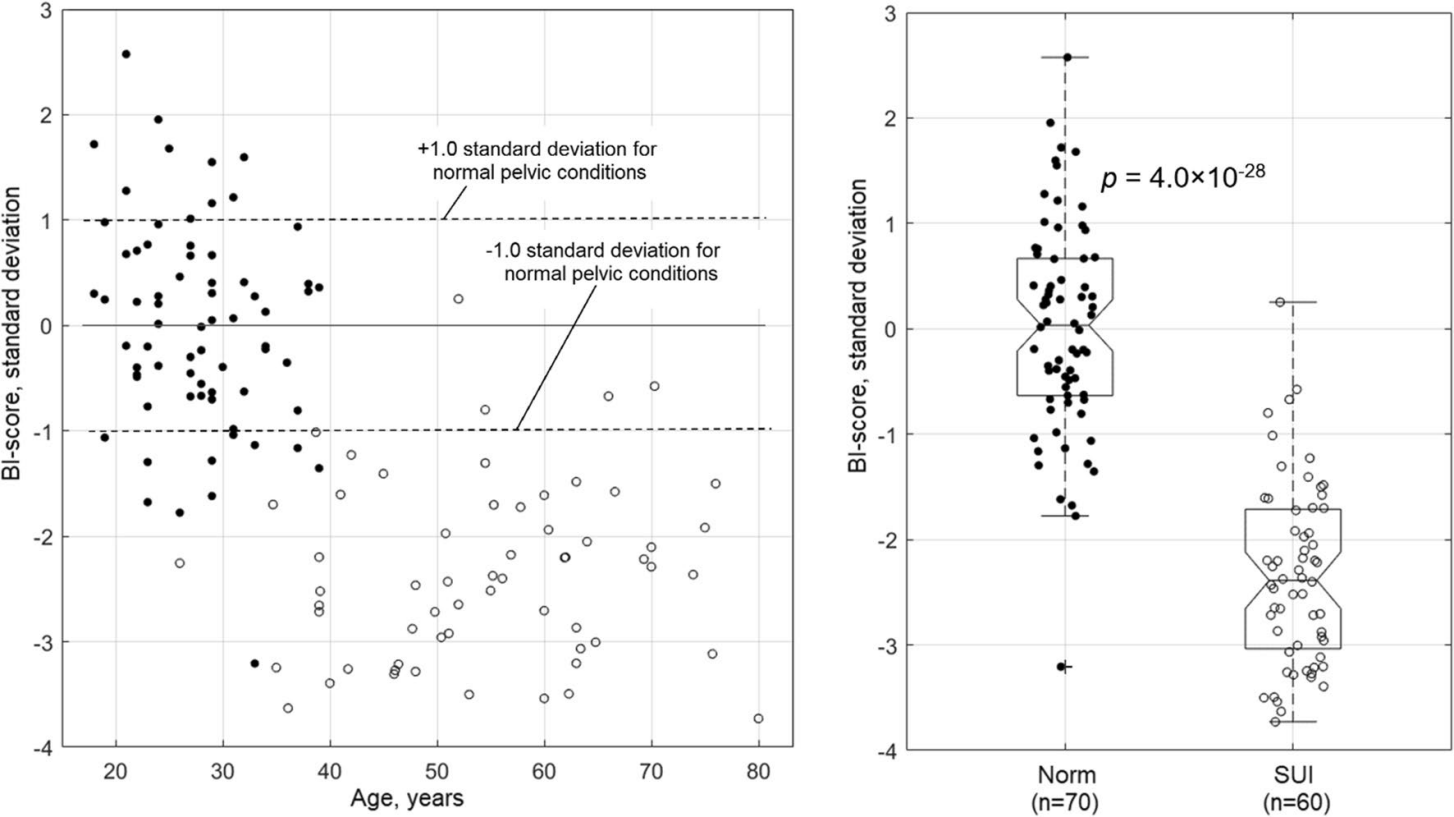
Biomechanical Integrity (*BI*)-score calculated for normal (*filled dots*) and stress urinary incontinence (*SUI*) (*open dots*) cases against patient age for 130 cases analyzed in this study (*left panel*). BI-score boxplots for normal and SUI cases (*right panel*)

All the BI-score data for 130 subjects analyzed here can be visualized on one graph as a function of the subject's age (see left panel in Fig. 4). The dashed lines show ± 1.0 standard deviation for normal pelvic conditions from the reference (zero) line. The right panel in Fig. 4 shows the same BI-score data in two boxplots for normal and SUI pelvic conditions. One may observe a significant separation between these two groups; the *t* test gives *p* = 4.0 × 10^–28^ for these two groups.

## Discussion

The BI-score is the composite score that consists of five components, as shown in Fig. 2. These five components bring different aspects of the biomechanical characterization of the pelvic floor. Owing to excluding the highly correlated original VTI parameters with *r* ≥ 0.85, the mutual correlation coefficients have an average value of *r* = 0.25, which is considered low. It is important to note that the tissue elasticity component integrates the tissue/structure elasticity for the 0- to 8-mm layer behind the vaginal walls from the depth comparative with the vaginal wall deformations in tests 1 and 3 (see Fig 2 and parameter interpretation in Egorov et al. [14]). The pelvic support component integrates the structure support from a depth of 5–45 mm, which is about the same as the vaginal wall deformations in test 2 (see Fig. 2 and parameter interpretation in Faulkner [20]).

Table 1 shows VTI parameter changes in SUI relative to the normal pelvic conditions. In SUI the elasticity parameters (−50.7% to −64.9%), the pelvic support parameters (−41.3% to −78.7%), and the muscle contraction parameters (−60.2% to −71.3%) are lower than in normal pelvic conditions. The muscle relaxation speed parameters, which have a negative sign because muscle force involuntarily goes down, are higher (131.5% to 152.8%) in SUI—relaxation develops faster.

The mean age and parity of the subjects in the normal and SUI groups are significantly different, which is the intended difference in the groups analyzed, because for the reference (zero line in the BI-score), we need a young population without SUI, which develops with age. As mentioned above, most of the *p* values of the VTI parameters (normal versus SUI) are < 1.0 × 10^−5^, and the *p* value for the BI-score is *p* = 4.0 × 10^–28^ for the two groups analyzed. It indicates that the data in these groups come from populations with unequal means and strong sensitivity to SUI. These results can be considered statistically significant validation for the BI-score sensitivity to SUI. Further, the SUI BI-score range can be subdivided into three zones—normal, transitional, and diseased—similar to what was suggested for the POP BI-score [11]. As with bone density measurement, monitoring patient progress with or without treatment is essential. Moreover, for this reason, it would be great to define the minimal clinically important difference in BI-score. The future research directions may also address:

1. BI-score use for monitoring a pelvic floor treatment outcome
2. Obtaining a periodic BI-score before a woman has symptoms
3. Recommendation for specific treatment based on the five components (e.g., treatment for elasticity is needed but not for relaxation or muscle mobility
4. Predictive capabilities of the BI-score for symptoms (e.g., a woman is less or more likely to develop some form of pelvic floor dysfunction)

These crucial questions are beyond this article.

The strength of this study is that the suggested BI-score covers biomechanical aspects of the pelvic floor, including tissue elasticity, pelvic support, muscle contraction, involuntary relaxation, and mobility. All these aspects usually deteriorate as the pelvic disease develops. This quantitative characterization can be used in diagnosing and monitoring pelvic conditions and selecting and justifying a treatment.

The weakness of this study is the absence of statistically significant results for possible variations in ethnicity and race, which must be the subject of future research. Also, thousands of new VTI examinations for normal pelvic floor conditions may adjust the mean and standard deviation values used in Eq. 1 for BI-score calculations. In addition, this was a cross-sectional study rather than a prospective longitudinal study, where disease processes can be assessed over time.

## Conclusion

Based on the analysis conveyed, five components, (tissue elasticity, pelvic support, pelvic muscle contraction, muscle relaxation, and muscle mobility) are vital for biomechanically characterizing the pelvic floor and particularly useful for describing biomechanical changes in women with and without SUI. All these components contribute to the integral parameter of the BI-score. Objectively measurable transformations of the pelvic tissues, support structures, and functions under different diseased conditions may be studied with the BI-score in future research and practical applications.

## References

[1] Grodstein F, Fretts R, Lifford K, Resnick N, Curhan G (2003). Association of age, race, and obstetric history with urinary symptoms among women in the Nurses’ Health Study. Am J Obstet Gynecol.

[2] Wu JM, Hundley AF, Fulton RG, Myers ER (2009). Forecasting the prevalence of pelvic floor disorders in U.S. Women: 2010 to 2050. Obstet Gynecol.

[3] Wu JM (2021). Stress incontinence in women. N Engl J Med.

[4] Haylen BT, de Ridder D, Freeman RM (2010). An International Urogynecological Association (IUGA)/International Continence Society (ICS) joint report on the terminology for female pelvic floor dysfunction. Int Urogynecol J.

[5] Minassian VA, Bazi T, Stewart WF (2017). Clinical epidemiological insights into urinary incontinence. Int Urogynecol J.

[6] Abufaraj M, Xu T, Cao C (2021). Prevalence and trends in urinary incontinence among women in the United States, 2005–2018. Am J Obstet Gynecol.

[7] Waetjen L, Xing G, Johnson W, Melnikow J, Gold E (2018). Factors associated with reasons incontinent midlife women report for not seeking urinary incontinence treatment over 9 years across the menopausal transition. Menopause.

[8] Diokno AC, Brock BM, Brown MB, Herzog AR (1986). Prevalence of urinary incontinence and other urological symptoms in the noninstitutionalized elderly. J Urol.

[9] Yalcin I, Bump RC (2003). Validation of two global impression questionnaires for incontinence. Am J Obstet Gynecol.

[10] DeLancey JO (1994). Structural support of the urethra as it relates to stress urinary incontinence: the hammock hypothesis. Am J Obstet Gynecol.

[11] Petros PE, Ulmsten UI (1990). An integral theory of female urinary incontinence. Experimental and clinical considerations. Acta Obstet Gynecol Scand Suppl.

[12] Falah-Hassani K, Reeves J, Shiri R, Hickling D, McLean L (2021). The pathophysiology of stress urinary incontinence: a systematic review and meta-analysis. Int Urogynecol J.

[13] Egorov V, van Raalte H, Lucente V, Sarvazyan A, Hoyte L, Damaser M (2016). Biomechanical characterization of the pelvic floor using tactile imaging. Biomechanics of the female pelvic floor.

[14] Egorov V, Shobeiri SA, Takacs P, Hoyte L, Lucente V, van Raalte H (2018). Biomechanical mapping of the female pelvic floor: prolapse versus normal conditions. Open J Obstet Gynecol.

[15] Egorov V, van Raalte H, Takacs P, Shobeiri SA, Lucente V, Hoyte L (2022). Biomechanical integrity score of the female pelvic floor. Int Urogynecol J.

[16] Egorov V, Sarvazyan AP (2008). Mechanical imaging of the breast. IEEE Trans Med Imaging.

[17] Egorov V, Ayrapetyan S, Sarvazyan AP (2006). Prostate mechanical imaging: 3-D image composition and feature calculations. IEEE Trans Med Imaging.

[18] Van Raalte H, Lucente V, Ephrain S (2016). Intra- and inter-observer reproducibility of vaginal tactile imaging. Urogynecology.

[19] Mcgill R, Tukey JW, Larsen WA (1978). Variations of box plots. Am Stat.

[20] Faulkner KG (2005). The tale of the T-score: review and perspective. Osteoporos Int.

